# A cohort study evaluating the risk of stroke associated with long-term exposure to ambient fine particulate matter in Taiwan

**DOI:** 10.1186/s12940-022-00854-y

**Published:** 2022-04-19

**Authors:** Pei-Chun Chen, Fung-Chang Sung, Chih-Hsin Mou, Chao W. Chen, Shan P. Tsai, Dennis H. P. Hsieh, Chung Y. Hsu

**Affiliations:** 1 Department of Public Health, China Medical University College of Public Health, 100 Jingmao Rd Sec. 1, Taichung, 406040 Taiwan; 2 Department of Health Services Administration, China Medical University College of Public Health, 100 Jingmao Rd Sec. 1, Taichung, 406040 Taiwan; 3 Management Office for Health Data, China Medical University Hospital, Taichung, Taiwan; 4 Department of Food Nutrition and Health Biotechnology, Asia University, Taichung, Taiwan; 5 University of Maryland Global Campus, Adelphi, MD USA; 6 School of Public Health, Texas A&M University, College Station, TX USA; 7 Department of Environmental Toxicology, University of California, Davis, CA USA; 8 Graduate Institute of Biomedical Sciences, China Medical University College of Public Health, Taichung, Taiwan

**Keywords:** Ischemic stroke, Hemorrhagic stroke, Particulate matter, Meteorological factors

## Abstract

**Background:**

Evidences have shown that the stroke risk associated with long-term exposure to particulate matter with an aerodynamic diameter of ≤2.5 μm (PM_2.5_) varies among people in North America, Europe and Asia, but studies in Asia rarely evaluated the association by stroke type. We examined whether long-term exposure to PM_2.5_ is associated with developing all strokes, ischemic stroke and hemorrhagic stroke.

**Methods:**

The retrospective cohort study consisted of 1,362,284 adults identified from beneficiaries of a universal health insurance program in 2011. We obtained data on air pollutants and meteorological measurements from air quality monitoring stations across Taiwan in 2010–2015. Annual mean levels of all environmental measurements in residing areas were calculated and assigned to cohort members. We used Cox proportional hazards models to estimate hazard ratio (HR) and 95% confidence interval (CI) of developing stroke associated with 1-year mean levels of PM_2.5_ at baseline in 2010, and yearly mean levels from 2010 to 2015 as the time-varying exposure, adjusting for age, sex, income and urbanization level.

**Results:**

During a median follow-up time of 6.0 years, 12,942 persons developed strokes, 9919 (76.6%) were ischemic. The adjusted HRs (95% CIs) per interquartile range increase in baseline 1-year mean PM_2.5_ were 1.03 (1.00–1.06) for all stroke, 1.06 (1.02–1.09) for ischemic stroke, and 0.95 (0.89–1.10) for hemorrhagic stroke. The concentration-response curves estimated in the models with and without additional adjustments for other environmental measurements showed a positively linear association between baseline 1-year mean PM_2.5_ and ischemic stroke at concentrations greater than 30 μg/m^3^, under which no evidence of association was observed. There was an indication of an inverse association between PM_2.5_ and hemorrhagic stroke, but the association no longer existed after controlling for nitrogen dioxide or ozone. We found similar shape of the concentration-response association in the Cox regression models with time-varying PM_2.5_ exposures.

**Conclusion:**

Long-term exposure to PM_2.5_ might be associated with increased risk of developing ischemic stroke. The association with high PM_2.5_ concentrations remained significant after adjustment for other environmental factors.

**Supplementary Information:**

The online version contains supplementary material available at 10.1186/s12940-022-00854-y.

## Introduction

The scientific statement from the American Heart Association indicated that exposure to particulate matter with an aerodynamic diameter of 2.5 μm or less (PM_2.5_) is associated with increased risk of cardiovascular morbidity and mortality, and that the risk associated with long-term exposure is likely to be stronger than that with short-term exposure [1]. While there is substantial evidence that long-term exposure to PM_2.5_ is associated with mortality from strokes, the reported risks of stroke are relatively weak and inconsistent [2–4]. Stroke is a heterogeneous disease with different etiology subtypes [5]. The major types of stroke are ischemic stroke and hemorrhagic stroke, and the associated risk factors are different between types of stroke. For example, although hypertension is a risk factor for both types of stroke, the strength of association and the population-attributable risk is larger for hemorrhagic stroke than for ischemic stroke [6, 7]. Diabetes, however, is a well-established risk factor of ischemic stroke but not hemorrhagic stroke [7]. Recently, two meta-analyses of cohort studies by Yuan et al. [8] and Alexeeff et al. [9] have shown a significant association between long-term exposure to PM_2.5_ and incidence of stroke. However, only four out of 16 studies [8] and five out of 14 studies [9] in the meta-analysis, respectively, provided data on ischemic stroke and hemorrhagic stroke separately, and the findings were inconclusive.

Assessment of risk by type of stroke may be particularly important for Asian populations, in which ethnic groups such as Chinese and Japanese exhibit a greater proportion of hemorrhagic stroke than do in Caucasians of European origin (proportions in community-based studies, 17–51% in Chinese vs. 6–20% in white populations) [10, 11]. Evidences have shown regional variations in the association between PM_2.5_ exposure and incidence of stroke [8, 9]. Few studies have been conducted in Asian populations to evaluate associations between long-term PM_2.5_ exposure and stroke risk by the stroke type [12–14]. PM_2.5_ exposure was associated with increased risk of ischemic stroke in all these studies, but results for the association with hemorrhagic stroke were inconsistent [12–14].

Furthermore, most studies focusing on long-term air pollution did not adjust for meteorological factors because the potential confounding effect is not yet clear. Meteorological factors have been linked to the risk of stroke in a short-term settings [15–17], whereas evidence for the associations in long-term settings remains sparse. A recent systematic review summarizing a few studies suggested that increased annual temperature was associated with increased rate of hospitalization for ischemic stroke and cardiovascular mortality [18]. Therefore, we conducted a population-based cohort study to evaluate whether long-term exposure to PM_2.5_ is associated with the risk of ischemic stroke and hemorrhagic stroke in the population of Taiwan, considering the potential confounding effect of multiple environmental exposures including ambient temperature.

## Methods and materials

### Study population

In Taiwan, over 99.5% of the people have enrolled in the National Health Insurance (NHI) program [19]. The computerized claims data of the NHI program comprise of medical records of all 23 million beneficiaries, and are managed and stored in the Health and Welfare Data Science Center, Ministry of Health and Welfare of Taiwan. The database consists of files of inpatient and outpatient cares, prescription drugs, and the Registry for Beneficiaries, between which linkages are conducted through encrypted personal identifications. Together, these files provided individual-level information on disease diagnoses, surgical procedures, medications, and demographics.

Using the claims data of NHI, we conducted a retrospective cohort study, in which a cohort of 4.5 million people was randomly selected from all NHI beneficiaries in 2011. For each cohort member, the index date was defined as the first date of enrollment for this study; if the first date was before January 1, 2011, we set the index date as January 1, 2011. Cohort members who were aged <20 years at the index date (*n* = 989,853), previously diagnosed with stroke (*n* = 314,331), or had invalid or missing data on survival status (*n* = 40) were excluded. Study subjects were considered as having a history of stroke if they had an outpatient or inpatient claim with a diagnosis of stroke before index date (International Classification of Diseases, 9th Revision, Clinical Modification [ICD-9-CM] codes 430.xx-438.xx). We also excluded subjects residing in offshore islands because the income data were unavailable (*n* = 5723), and those residing in administrative subdivisions without air quality monitoring station (*n* = 1,827,769). A total of 1,362,284 adults were included in our analysis.

In Taiwan, there are 22 administrative divisions in terms of special municipality, county and provincial city directed by the central government. The 22 divisions are further divided into 368 administrative subdivisions for which the official terms were city, township, or district. The study population, monitoring stations and the administrative subdivisions that were included and excluded from our data analysis are shown in Fig. S1 in the Supplementary Material in the Additional file 1. In Taiwan, nearly 80% of the total 76 air quality monitoring stations are set up in cities and counties with high population density (general stations). The remaining stations are installed in the metropolitan areas with high traffic volume (traffic stations), industrial areas (industrial stations), the national parks (national park stations), and the areas with less pollution (background stations and other stations for specific purpose) [20]. The study subjects included in our analysis were residents of areas with air quality monitoring stations. Therefore, they were more likely to live in more urbanized areas with higher income (Table SI). However, distributions of age, sex, and comorbidities were similar between residents included and excluded in this study (Table SI). The Research Ethics Committee at China Medical University and Hospital approved the study protocol (CRREC-107-021(CR-3)).

### Assessment of long-term PM_2.5_ exposure

The Environmental Protection Administration of Taiwan has established air quality monitoring stations since 1990s throughout the whole island to measure hourly air pollutants, including sulfur dioxide (SO_2_, ppb), particulate matter with an aerodynamic diameter ≤ 10 μm (PM_10_, μg/m^3^), carbon monoxide (CO, ppm), nitrogen oxides (NO_x_, ppb), ozone (O_3_, ppb), and nitrogen dioxide (NO_2_, ppb). As of the end of 2005, there were 76 monitoring stations, and these stations have started monitoring hourly measures of PM_**2.5**_ since 2005 [20]. Previous studies have shown that the stroke risk associated with PM_2.5_ exposure in recent 1- to 5-year was stronger than the exposure in earlier period (i.e., 5 years before baseline) [21, 22]. Therefore, we used PM_2.5_ measured from 2010 to 2015 at the administrative subdivisions (i.e., city, township, or district) as the proxy for individual exposure because the address information in the NHI claims data is limited to the subdivision level for the protection of patient privacy. We calculated annual average PM_2.5_ concentrations using the hourly data by administrative subdivision with at least one monitoring station, and assigned the PM_2.5_ levels to study subjects by the administrative subdivision they lived in. We used only the measurements collected from stations available in the respective administrative subdivision. In the area with more than one monitoring stations, we used the mean level of all stations. Measures of PM_2.5_ were available for all study subjects in 2010, and the percentage of people with missing values ranged from 0.6 to 9.2% from 2011–2015 (Table S II). Subjects with missing values were excluded from the regression analysis in which PM_2.5_ was considered time-varying exposures. We also inspected missing values among monitoring stations. Of all stations from 2010 to 2015, only 1.0% station-years (*n* = 4) had missing values for more than 20% of a year (i.e., missing data on daily exposure measurement for more than 73 of the 365 days), and 1.6% station-years (*n* = 7) had missing values for more than 10% of a year (i.e., 36 of the 365 days).

### The potential confounders and comorbidity

We identified the potential confounders based on literature review. Data on demographics and socioeconomic status that have been associated with the stroke risk [23], available in the claims data for this study included age, sex, urbanization level of the residential area, and per capita disposable income. The income level was estimated using per Capita Disposable Income reported by Directorate General of Budget, Accounting and Statistics, Executive Yuan, Taiwan, on the yearly basis [24]. We used the classification scheme developed by Liu et al. to determine the urbanization level of the administrative subdivision where a study subject registered as residence [25]. In brief, all cities, townships, and districts in Taiwan were classified into 7 urbanization levels according to a score computed based on population density (people/km^2^), proportion of people with a college or higher level of education, proportion of elderly and agricultural population, and the number of physicians per 100,000 people. Level 1 is the most urbanized. Levels 4 to 7 were combined because of the small number of subjects in these categories.

In addition to PM_2.5_, data of NO_2_, NO_x_, and SO_2_ were also used in this study, as several studies have associated these air pollutants with increased risks of stroke and cardiovascular disease [26–31]. Long-term exposure to ambient temperature also has been linked to increased hospitalization for ischemic stroke and cardiovascular risk [18, 32]. Therefore, we considered meteorological conditions as potential risk factors associated with stroke, focusing on temperature. Data on all air pollutants, temperature and relative humidity were obtained from the same air quality monitoring stations where the PM_2.5_ concentrations were collected (Fig. S1). For all the air pollutants, data from all 74 monitoring stations were used, excluding 2 stations in offshore islands (Fig. S1). Temperature and relative humidity were unavailable at 2 stations in 2010, resulting missing values for 38,583 subjects (or 2.8% of all study subjects) (Table S II). Subjects included in our main exposure (PM_2.5_) analysis and those with temperature data were similar in distributions of socio-demographic characteristics and comorbidities at baseline (number of subjects, 1,362,284 and 1,323,701, respectively, Table S III). The data processing procedure for all environmental variables including temperature was similar to that for PM_2.5_. Annual mean levels of these environmental variables measured during 2010–2015 were assigned to the study subjects based on their registered residence, by the city/township /district level.

From the claims data, we identified several comorbidities known as risk factors of stroke, including diabetes mellitus (ICD-9-CM code 250), hypertension (ICD-9-CM codes 401–405), hyperlipidemia (ICD-9-CM code 272), coronary artery disease (ICD-9-CM codes 410–414), chronic obstructive pulmonary disease (ICD-9-CM codes 491, 492, 496) and atrial fibrillation (ICD-9-CM code 427.3). ICD-10 version was not applicable in this study because in Taiwan, ICD-9 version had been adapted until the end of 2015. A comorbid condition was defined by at least one inpatient or two outpatient claims for the disease diagnosed within two years before the index date. Evidence has shown that the pathophysiological mechanisms linking long-term PM_2.5_ exposure and cardiovascular health involved inflammation, atherosclerosis, and changes in vascular functions [1]. Therefore, the comorbidities were not adjusted for in the regression analysis, as they might more likely be the mediators rather than confounders. However, we performed stratified analyses by these comorbidities to observe whether the associations were homogenous in the patient subgroups.

### Health outcomes

Study subjects were followed up to observe the occurrence of stroke, which was considered to be present if study subjects had a hospital admission with a primary discharge diagnosis of stroke (ICD-9-CM code 431, 432, 433, 434, 436). Follow-up person-years were calculated for each subject from the index date until the diagnosis of stroke, or until censored because of withdrawal from the insurance coverage or death, or December 31, 2016. We assessed survival status and date of death from the Registry for Cause of Death, and classified cases of hemorrhagic stroke (ICD-9-CM codes 431–432) and ischemic stroke (ICD-9-CM codes 433, 434, 436)。.

### Statistical analysis

Baseline characteristics of study subjects were described using mean (standard deviations, SD) for continuous variables and the number of subjects (percentages) for categorical variables. We presented distributions of annual mean levels of temperature, humidity and air pollutants from 2010 to 2015, and listed the missing values, mean, median, quartile 1, quartile 3, and minimum and maximum values. Cox proportional hazards models were applied to evaluate the association between long-term PM_**2.5**_ exposure and risks of all strokes, ischemic stroke, and hemorrhagic stroke. We performed two sets of analyses, using different time windows of exposures: mean PM_2.5_ level in 2010, and yearly mean levels of PM_2.5_ from 2010 to 2015 treated as a time-varying exposure in the Cox models. To take into account residence changes in the time-varying Cox proportional hazard models, we updated the registered residence locations yearly from 2010 to the end of follow-up. The yearly mean level of PM_2.5_ was assigned according to the updated location of residence for each individual. In each set of analyses, hazard ratios (HRs) and 95% confidence intervals (CIs) were reported per interquartile range (IQR) increase in PM_2.5_ (single-exposure model). We performed two models with increasing level for adjustment. Model 1 was adjusted for age (in continuous scale, years) and sex (men, women). In Model 2, our main model, we additionally adjusted for income level (<=272,470, 272,471-273,351, 273,351-311,566, >311,566, New Taiwan dollars per year) and the urbanization level (levels 1, 2, 3, 4–7). To explore the effect of other environmental exposures, the single-exposure model was repeated by substituting PM_2.5_ with NO_2_, NO_x_, SO_2_, O_3_, CO, temperature or relative humidity. To assess if the association between PM_2.5_ and risk of stroke changes after controlling for other environmental exposures and vice versa, we performed two-exposure models to assess environmental variables significantly associated with developing stroke in the single-exposure models. In the two-exposure models, we included NO_2_ but not NO_x_, as evidence has shown a consistent association between NO_2_ and risk of stroke [2, 31]. In all the single- and two-exposure models, the environmental exposure variables were entered as linear terms (per IQR increase). The proportional hazards assumption was examined by including product terms between the environmental variables and a function of follow-up time, and the results showed no violation of the assumption. To assess correlations between environmental exposures in two-exposure models, we calculated Spearman correlation coefficients yearly from 2010 to 2015.

We evaluated the exposure-response relationship between PM_2.5_ levels and incidence of all stroke, ischemic stroke and hemorrhagic stroke using restricted cubic splines with three knots located at the 10th, 50th, and 90th percentiles of the distributions of PM_2.5_ (R package rms and survival) in Cox regression analysis [33, 34]. The first model, our main model, was adjusted for all socio-demographic variables including age, sex, income level and urbanization level. We then performed 5 models based on the main model, additionally adjusting for NO_2_, SO_2_, O_3_, CO, or temperature that were significantly associated with stroke in single-exposure models, to observe the shape of the association between PM_2.5_ and stroke considering other environmental exposures. In addition, we performed a sensitivity analysis, in which natural cubic splines with two degrees of freedom were fitted to evaluate the consistency of shapes of dose-response relationships.

Stratified analyses were performed to assess the potential effect modification by comorbidities. The interaction terms were tested using likelihood ratio test. We performed two sensitivity analyses to assess the impact of misclassification of residence because a portion of study subjects may not reside at their registered residence. First, we used the estimated current residence instead of registered residence in the Cox regression analysis. The current residence was estimated using the algorithm developed by Ku et al., based on sequentially considering several locations such as locations of outpatient clinic visit for low respiratory tract infections, locations of primary physician clinics visit, and geographical proximity between registered residence and the locations of access to primary healthcare [35]. Second, we performed data analysis excluding people whose location of registered residence was inconsistent with that of their NHI registration. In Taiwan, areas where people register in the NHI program are usually locations of their residences or employments. Data processing and statistical analyses were performed using SAS Version 9.4 (SAS Institute, Inc., Cary, NC, USA) and R version 4.1.0. A two-tailed *p* value <0.05 was considered statistically significant.

## Results

**Fig. 1:**
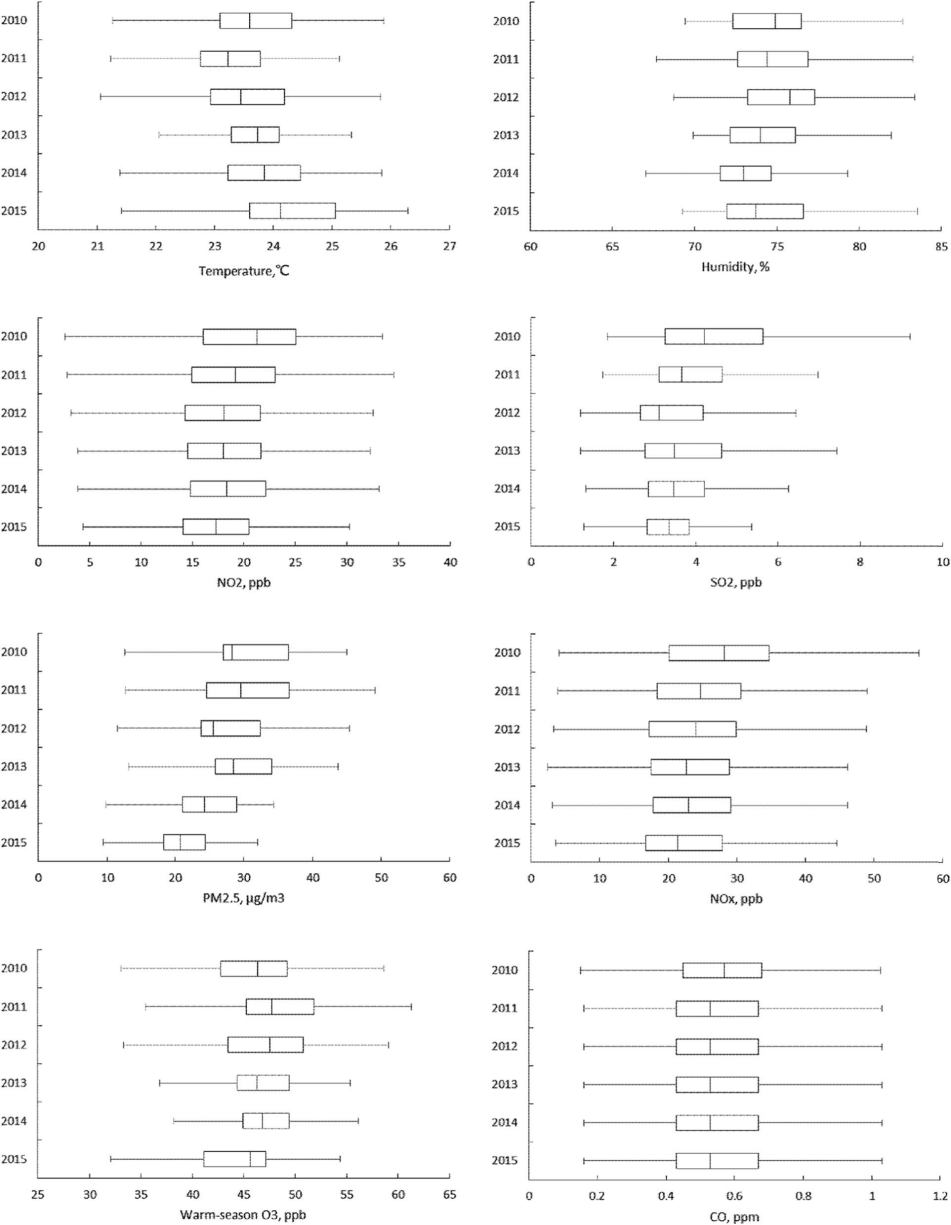
Annual mean levels of air pollutants, temperature and humidity assigned for each subject by calendar year, 2010–2015, Taiwan

A total of 1,362,284 adults were included in data analyses (Table 1). The mean age (SD) of the study population at index date was 44.0 (15.2) years, with 48.4% of men. During a median follow-up time of 6.0 years, 12,942 participants developed strokes, resulting in an incidence rate of 1.6 per 1000 person-years (Table 1). Of all stroke cases, 9919 (76.6%) were ischemic and 3023 (23.4%) were hemorrhagic. The annual mean (SD) concentrations of PM_2.5_ decreased from 30.4 (7.2) μg/m^3^ in 2010 to 21.1 (4.5) μg/m^3^ in 2015 (Fig. 1 and Table S II). However, the median levels and IQR of PM_2.5_ varied during the study period: the IQRs were 9.6 μg/m^3^ in 2010 and 12.1 μg/m^3^ in 2011, then decreased to 8.7 μg/m^3^ in 2012, 8.3 μg/m^3^ in 2013, 7.9 μg/m^3^ in 2014, and 6.1 μg/m^3^ in 2015. Mean levels of SO_2_ and NO_2_ were also in downward trends. The annual mean (SD) temperature ranged from 23.2 (1.4)°C to 24.2 (1.4)°C during the 6-year period, and was slightly higher in 2013–2015 than in 2010–2012 [mean (SD), 23.9 (1.4)°C vs. 23.4 (1.5)°C, *p* < 0.001].

**Table 1:** Descriptive characteristics of the study cohort at baseline in 2011 and the health outcomes

	Study cohort, *N* = 1,362,284	
Variable	n	%
Age, years		
20–44	735,384	54.0
45–64	495,520	36.4
65–74	82,146	6.0
75+	49,234	3.6
Mean (SD)	44.0	(15.2)
Men	659,112	48.4
Urbanization level		
1 (most urbanized)	359,435	26.4
2	606,694	44.5
3	232,680	17.1
4–7	163,475	12.0
Income level, Taiwan dollars^a^		
< =272,470	432,566	31.8
272,471–273,351	369,701	27.1
273,351–311,566	338,977	24.9
> 311,566	221,040	16.2
Comorbidity^b^		
Diabetes	80,181	5.9
Hypertension	171,447	12.6
Hyperlipidemia	113,536	8.3
Coronary artery disease	42,894	3.2
Chronic obstructive pulmonary disease	55,700	4.1
Atrial fibrillation	4054	0.3
Outcomes and follow-up		
Person-years at risk		
Total	8,019,765	
Mean (Median)	5.9 (6.0)	
Number of events (Incidence)^c^		
Total stroke	12,942 (1.6)	
Ischemic stroke	9919 (1.2)	
Hemorrhagic stroke	3023 (0.4)	

Abbreviation: *N* number of study subjects, *SD* standard deviation

Data are n (%) unless otherwise noted

^a^per Capita Disposable Income

^b^Patients with at least one inpatient or two outpatient claims with a diagnosis of the comorbid conditions in two years before the index date

^c^Incidence per 1000 person-years

In the single-exposure main models, after adjusting for age, sex, income level and urbanization level, risks of developing total stroke and ischemic stroke increased with air pollutants, except warm-season O_3_ (Table 2). HRs (95% CIs) of ischemic stroke per IQR increase in 1-year PM_2.5_ levels at baseline decreased from 1.10 (1.07–1.13) in the age- and sex- adjusted model to 1.06 (1.02–1.09) after additionally controlling for levels of income and urbanization. An elevated temperature was associated with a slightly increased risk of total stroke and ischemic stroke, with adjusted HRs (95% CI) of 1.02 (1.00–1.04) and 1.03 (1.01–1.05), respectively. No association with relative humidity was observed. We found no association between most of the environmental exposures and risk of developing hemorrhagic stroke except positive associations with NO_x_ and CO at a borderline significance level. On the other hand, O_3_ was associated with reduced risk (HRs 0.92, 95% CI 0.88–0.97) after adjusting for all covariates. The main findings in time-varying Cox models showed similar relationships between environmental exposures and stroke risks, except that a weak inverse association appeared between PM_2.5_ and hemorrhagic stroke after adjusting for all covariates (Table 2).

**Table 2:** Hazard ratios (95% confidence intervals) of stroke associated with per interquartile-range increase in air pollutant, temperature and humidity

	1-year level at baseline^a^			Air pollution level as a time-varying covariate^d^	
	IQR	Model 1^b^	Model 2 (Main model)^c^	Model 1^b^	Model 2 (Main model)^c^
Total Stroke					
PM_2.5,_ μg/m^3^	9.6	1.07 (1.04–1.09)	1.03 (1.00–1.06)	1.07 (1.06–1.09)	1.03 (1.01–1.05)
SO_2_, ppb	2.4	1.06 (1.03–1.08)	1.03 (1.00–1.05)	1.05 (1.04–1.07)	1.03 (1.02–1.05)
NO_x_, ppb	14.5	0.97 (0.95–0.98)	1.04 (1.02–1.06)	0.97 (0.96–0.98)	1.04 (1.02–1.05)
NO_2_, ppb	9.0	0.94 (0.92–0.96)	1.04 (1.00–1.07)	0.95 (0.93–0.96)	1.04 (1.02–1.07)
Warm-season O_3_^e^, ppb	6.5	1.05 (1.02–1.07)	0.98 (0.96–1.00)	1.04 (1.02–1.05)	0.97 (0.96–0.99)
CO, ppm	0.2	0.97 (0.95–0.98)	1.04 (1.02–1.06)	0.97 (0.96–0.98)	1.03 (1.01–1.04)
Temperature, °C	1.2	1.05 (1.03–1.06)	1.02 (1.00–1.04)	1.05 (1.04–1.06)	1.02 (1.01–1.03)
Relative humidity, %	4.1	1.01 (0.996–1.03)	1.00 (0.97–1.02)	1.00 (0.99–1.01)	0.99 (0.97–1.00)
Ischemic stroke					
PM_2.5,_ μg/m^3^	9.6	1.10 (1.07–1.13)	1.06 (1.02–1.09)	1.11 (1.09–1.13)	1.05 (1.03–1.08)
SO_2_, ppb	2.4	1.08 (1.05–1.10)	1.04 (1.01–1.07)	1.08 (1.06–1.10)	1.05 (1.03–1.07)
NO_x_, ppb	14.5	0.96 (0.95–0.98)	1.04 (1.01–1.06)	0.97 (0.96–0.98)	1.04 (1.02–1.06)
NO_2_, ppb	9.0	0.94 (0.92–0.97)	1.05 (1.01–1.09)	0.95 (0.94–0.97)	1.05 (1.03–1.08)
Warm-season O_3_^e^, ppb	6.5	1.07 (1.05–1.10)	1.00 (0.97–1.02)	1.06 (1.04–1.08)	0.99 (0.97–1.01)
CO, ppm	0.2	0.96 (0.95–0.98)	1.04 (1.01–1.06)	0.97 (0.96–0.98)	1.03 (1.01–1.04)
Temperature, °C	1.2	1.06 (1.04–1.08)	1.03 (1.01–1.05)	1.06 (1.05–1.07)	1.03 (1.02–1.04)
Relative humidity, %	4.1	1.01 (0.99–1.03)	0.99 (0.97–1.02)	0.99 (0.98–1.01)	0.98 (0.97–1.00)
Hemorrhagic stroke					
PM_2.5,_ μg/m^3^	9.6	0.97 (0.92–1.01)	0.95 (0.89–1.10)	0.96 (0.93–1.00)	0.94 (0.91–0.98)
SO_2_, ppb	2.4	0.98 (0.94–1.03)	0.98 (0.93–1.03)	0.97 (0.94–1.01)	0.97 (0.94–1.01)
NO_x_, ppb	14.5	0.98 (0.95–1.01)	1.04 (1.00–1.08)	0.98 (0.95–1.00)	1.04 (1.00–1.07)
NO_2_, ppb	9.0	0.93 (0.89–0.98)	1.02 (0.95–1.09)	0.94 (0.91–0.97)	1.01 (0.97–1.06)
Warm-season O_3_^e^, ppb	6.5	0.97 (0.93–1.02)	0.92 (0.88–0.97)	0.97 (0.94–1.00)	0.93 (0.90–0.96)
CO, ppm	0.2	0.98 (0.95–1.01)	1.04 (1.00–1.08)	0.98 (0.96–1.00)	1.03 (1.00–1.05)
Temperature, °C	1.2	0.98 (0.97–1.03)	0.99 (0.96–1.02)	1.00 (0.98–1.02)	0.99 (0.96–1.01)
Relative humidity, %	4.1	1.03 (0.99–1.07)	1.01 (0.96–1.05)	1.02 (0.99–1.04)	1.00 (0.97–1.03)

Abbreviations: *CO* carbon monoxide, *IQR* interquartile range, *NO*_*2*_ nitrogen dioxide, *NO*_*x*_ nitrogen oxides, *O*_*3*_ ozone, *PM*_*2.5*_ particulate matter of <2.5 μm in diameter, *SO*_*2*_ sulfur dioxide

^a^There were no missing values for the covariates in models 1 and 2. There were missing values for temperature, relative humidity and O_3_. Therefore, Numbers of subjects included in the analysis for each environmental exposure (single-pollutant model) were 1,323,701 for temperature and relative humidity, 1,362,284 for PM_2.5_, SO_2_, NO_x_, NO_2_ and CO, and 1,344,570 for O_3_

^b^Model 1 was adjusted for age and sex

^c^Model 2 was adjusted for age, sex, income, and urbanization level

^d^Obervations with missing values were excluded from the time-vary Cox proportional hazard models. Numbers of observations included in the models were: 7,569,935 for temperature and relative humidity, 7, 797,277 for PM_2.5_, SO_2_, NO_x_, NO_2_; 7,637,106 for O_3_; and 7,791,276 for CO.

^e^April-October daily maximum 8-h ozone concentrations

In the two-exposure models for 1-year air pollution exposure at baseline, the positive association between PM_2.5_ concentrations and risk of ischemic stroke remained statistically significant after adjusting for all environmental exposures except CO. However, the HRs for NO_2_ and temperature decreased to a non-significant level after controlling for PM_2.5_ (Table 3). In the analysis with time-varying environmental exposures, additional adjustment for SO_2_ and temperature resulted in a weakened, non-signification association between PM_2.5_ concentrations and risk of ischemic stroke (Table S IV). HRs for all environmental exposures remained at similar strength after controlling for PM_2.5_ (Table S IV). Overall, annual mean PM_2.5_ levels had positive correlations with other environmental exposures during 2010–2015 (Fig. S II). The Spearman correlation coefficients with temperature (from 0.51–0.70) were higher than that with other air pollutants. The correlation coefficients with SO_2_ were weak to moderate (from 0.30–0.54), and with other air pollutants were mostly weak.

**Table 3:** Hazard ratios (95% confidence intervals) of stroke associated with 1-year air pollution exposure at baseline (per IQR increase) in the two-exspoure models

	Single-exposure model^a^	Two-exposure models^a^, additionally adjusted for					
		PM_2.5,_ μg/m^3^	SO_2,_ ppb	NO_2_, ppb	Warm-season O_3_, ppb	CO, ppm	Temperature°C
Total Stroke							
PM_2.5,_ μg/m^3^	1.03 (1.00–1.06)	–	1.02 (0.99–1.06)	1.02 (0.98–1.05)	1.05 (1.02–1.09)	1.01 (0.98–1.05)	1.03 (1.00–1.07)^b^
SO_2,_ ppb	1.03 (1.00–1.05)	1.02 (1.00–1.05)^b^	–	1.02 (0.99–1.05)	1.02 (1.00–1.05)^b^	1.02 (0.99–1.04)	1.02 (1.00–1.05)
NO_2_, ppb	1.04 (1.00–1.07)	1.03 (0.99–1.07)	1.03 (0.99–1.07)	–	1.03 (0.99–1.06)	1.02 (0.98–1.06)	1.03 (1.00–1.07)^b^
Warm-season O_3_, ppb	0.98 (0.96–1.00)	0.96 (0.94–0.99)	0.98 (0.96–1.01)	0.98 (0.96–1.01)	–	0.98 (0.95–1.00)	0.98 (0.95–1.00)
CO, ppm	1.04 (1.02–1.06)	1.02 (1.00–1.04)^b^	1.02 (1.00–1.04)^b^	1.02 (1.00–1.04)^b^	1.02 (1.00–1.0.4)	–	1.03 (1.01–1.05)
Temperature, °C	1.02 (1.00–1.04)	1.00 (0.98–1.03)	1.00 (0.98–1.02)	1.01 (0.98–1.03)	0.99 (0.97–1.02)	1.02 (0.99–1.04)	–
Ischemic stroke							
PM_2.5,_ μg/m^3^	1.06 (1.02–1.09)	–	1.04 (1.01–1.08)	1.05 (1.01–1.09)	1.07 (1.03–1.11)	1.03 (0.99–1.08)	1.06 (1.02–1.10)
SO_2,_ ppb	1.04 (1.01–1.07)	1.03 (1.00–1.06)	–	1.03 (1.00–1.07)	1.04 (1.01–1.07)	1.03 (1.00–1.06)	1.04 (1.01–1.07)
NO_2_, ppb	1.05 (1.01–1.09)	1.02 (0.98–1.07)	1.02 (0.98–1.07)	–	1.04 (1.00–1.08)	1.02 (0.98–1.06)	1.04 (0.99–1.08)
Warm-season O_3_, ppb	1.00 (0.97–1.02)	0.98 (0.95–1.00)	1.01 (0.98–1.03)	1.00 (0.97–1.03)	–	1.00 (0.97–1.02)	0.99 (0.97–1.02)
CO, ppm	1.04 (1.01–1.06)	1.02 (1.00–1.05)^b^	1.03 (1.01–1.05)	1.03 (1.01–1.05)	1.03 (1.01–1.05)	–	1.04 (1.02–1.07)
Temperature, °C	1.03 (1.01–1.05)	1.01 (0.98–1.04)	0.99 (0.97–1.02)	1.00 (0.97–1.03)	0.99 (0.97–1.02)	1.02 (0.99–1.05)	–
Hemorrhagic stroke							
PM_2.5,_ μg/m^3^	0.95 (0.89–1.10)	–	0.95 (0.89–1.02)	0.93 (0.87–1.00)	0.99 (0.93–1.06)	0.95 (0.88–1.02)	0.95 (0.88–1.01)
SO_2,_ ppb	0.98 (0.93–1.03)	0.99 (0.94–1.05)	–	0.96 (0.90–1.02)	0.96 (0.91–1.01)	0.98 (0.93–1.04)	0.98 (0.93–1.03)
NO_2_, ppb	1.02 (0.95–1.09)	1.05 (0.98–1.13)	1.05 (0.97–1.13)	–	0.99 (0.93–1.06)	1.03 (0.96–1.11)	1.03 (0.95–1.11)
Warm-season O_3_, ppb	0.92 (0.88–0.97)	0.92 (0.88–0.97)	0.92 (0.87–0.96)	0.92 (0.88–0.97)	–	0.92 (0.88–0.97)	0.92 (0.88–0.97)
CO, ppm	1.04 (1.00–1.08)	1.00 (0.96–1.05)	0.99 (0.96–1.03)	0.98 (0.95–1.02)	0.98 (0.95–1.02)	–	0.99 (0.94–1.03)
Temperature, °C	0.99 (0.96–1.02)	0.99 (0.94–1.04)	1.00 (0.96–1.05)	1.02 (0.96–1.07)	1.00 (0.96–1.05)	0.99 (0.94–1.04)	–

Abbreviations: *CO* carbon monoxide, *IQR* interquartile range, *NO*_*2*_ nitrogen dioxide, *NO*_*x*_ nitrogen oxides, *PM*_*2.5*_ particulate matter of <2.5 μm in diameter, *SO*_*2*_ sulfur dioxide

^a^The model was adjusted for age, sex, income, and urbanization level

^b^Borderline significance (*P near* 0.05)

**Fig. 2:**
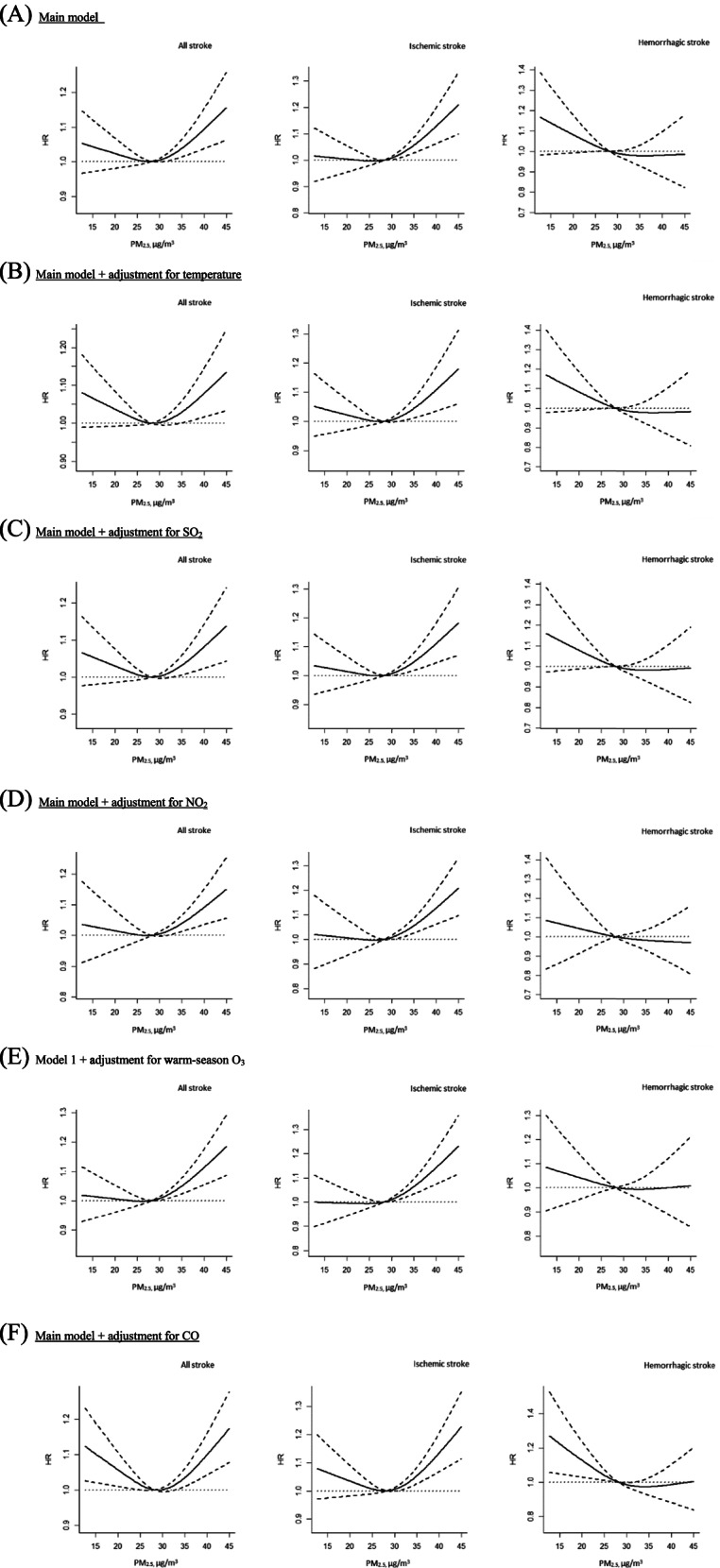
Hazard ratios (HRs) of all stroke, ischemic stroke and hemorrhagic stroke by long-term exposure to 1-year exposure of PM_2.5_ at baseline without additional adjustment (**A**) and with additional adjustment for temperature (**B**), SO_2_ (**C**), NO_2_ (**D**), Warm-season O_3_ (**E**), and CO (**F**)

Fig. 2 illustrates the concentration-response relationship between 1-year PM_2.5_ exposure at baseline and incidence of all stroke, ischemic stroke and hemorrhagic stroke in the models with or without adjustment for other air pollutants. In all models, we found a V-shaped association with ischemic stroke positive and significant at concentrations greater than 30 μg/m^3^, under which no evidence of association was observed (Fig. 2, (A) to (F)). There was no indication of threshold for concentrations greater than 30 μg/m^3^. In contrast, there was a statistically non-significant decreased HRs of hemorrhagic stroke at concentrations at about 25 μg/m^3^ and lower. However, such an inverse association no longer existed after controlling for NO_2_ or O_3_ (Fig. 2, (D) and (E)). The sensitivity analysis where natural cubic spline was fitted revealed similar results (Fig. S III).

We found similar shape of the concentration-response association in the analysis of Cox regression with the environmental exposures as time-varying covariates (Fig. S IV). The slope for the association of PM_2.5_ and ischemic stroke at concentrations >30 μg/m^3^ was less steep after adjusting for temperature, but the association remained statistically significant (Fig. S IV, (B)). The inverse association between PM_2.5_ and hemorrhagic stroke was attenuated to a statistically non-significant level after controlling for NO_2_ or O_3_ (Fig. S IV, (D) and (E)).

The dose-response curves with temperature indicated a progressively increased risk of ischemic stroke with elevating temperature with steeper slope at temperatures above approximately 24 °C (Fig. S V). In contrast, there was an inverse association between temperature and risk of hemorrhagic stroke with steeper slope at low temperatures, but the HRs were not statistically significant across the range of temperatures.

Stratified analyses showed that the strength of ischemic stroke risks associated with per IQR increment of PM_2.5_ changed little in all patient groups between those with and without comorbidities, except patients with diabetes among whom the HR was weakened to 1.01 (Fig. VI (A) (P for interaction >0.05 for all comorbidities). The precision of the HRs decreased in patient groups with comorbidities probably because of the smaller number of subjects in the patient groups than their counterparts without comorbidity. In all the patient groups stratified by comorbidities, there was no statistically significant association between PM_2.5_ levels and risk of developing hemorrhagic stroke (Fig. S VI (B)).

The sensitivity analysis estimated exposures in current residence areas, instead of registered residence areas, and showed that findings on stroke risks (Table S V) were similar to findings in the main analysis of the single-exposure model (Table 2). In the analysis restricted to people whose locations of registered residence were consistent with that of their NHI registration, the HRs for all air pollutants, temperature and relative humidity were also similar to that in our main analysis of single-exposure model (Table S VI).

## Discussion

In this population-based cohort study, elevated ambient PM_2.5_ levels were found to be associated with moderately increased risk of incidence of total strokes and ischemic stroke after controlling for study population characteristics. Our observations indicated a linear concentration-response relationship with ischemic stroke when PM_2.5_ level was greater than 30 μg/m^3^. The shape of the association was similar in models additionally controlling for other air pollutants or temperature. We found an indication of an inverse association between PM_2.5_ and hemorrhagic stroke, but the association no longer existed after adjustment for NO_2_ or O_3_.

Epidemiological studies generally reported a stronger association between long-term PM_2.5_ exposure and risk of ischemic stroke risk than that of hemorrhagic stroke. Our findings were in line with that in recent studies conducted in Hong-Kong Chinese cohort and Danish Nurse cohort, which showed that PM_2.5_ exposure was associated with increased risk of ischemic stroke, but not hemorrhagic stroke [14, 36]. Huang et al. [12] and Shin et al. [37] reported risks for both stroke types increased with the PM_2.5_ level, but the association with hemorrhagic stroke was weaker. An analysis based on the US Health Professionals Follow-up Study, on the contrary, indicated no association between long-term PM_2.5_ exposure and the overall and ischemic stroke, but there was a statistically non-significant association between PM_2.5_ and increased incidence of hemorrhagic stroke [38]. Discrepant findings among studies may reflect variations in characteristics of study populations, methods of exposure assessments, and the covariates considered. For example, evidence has shown a positive association between road traffic noise and risk of stroke, independent [39] or not independent [40] of air pollution. However, most studies, including ours, did not consider the potential confounding effect of noise exposure in assessing stroke risk associated with PM_2.5_ exposure. Variations in types and levels of environmental exposures, such as road traffic noise, among populations may partly explain the inconsistent findings among studies. In addition, variation in the PM_2.5_ levels could also explain the different findings across studies. In the cohort study from China-PAR project by Huang et al. [12], the average PM_2.5_ level over 16 years was 64.9 μg/m^3^. It was greater than the 1-year mean level of PM_2.5_ of 35.8 μg/m^3^ in the Hong-Kong Chinese cohort [14], or 19.7 μg/m^3^ in the Danish Nurse cohort [36], and 30.4 μg/m^3^ in 2010 or 21.1 μg/m^3^ in 2015 in the present study.

Whether there is a possible threshold of PM_2.5_ concentration associated with the stroke risk remains inconclusive. A large prospective cohort study and meta-analysis from the European Study of Cohorts for Air Pollution Effects (ESCAPE) demonstrated that among people with PM_2.5_ level < 25 μg/m^3^ (the European limit value), long-term exposure to PM_2.5_ with 5 μg/m^3^ increment was associated with significantly elevated risk of cerebrovascular events [3]. A pooled analysis of six European cohorts also showed that there was no threshold of PM_2.5_ relating to the stroke risk, and the slope of the concentration-response curve was steeper at low concentrations than high concentrations [31]. The Danish Nurse cohort study has suggested a threshold level of PM_2.5_ around 20 μg/m^3^, and found no elevated risk of ischemic stroke associated with further increased PM_2.5_ level [36]. On the contrary, the US-based Health Professionals Follow-up Study found no association between PM_2.5_ concentrations and the stroke incidence, and the estimated HRs were not statistically significant across the whole range of PM_2.5_ concentrations [38]. In our study, however, there is little evidence demonstrating an increased ischemic stroke risk at the PM_2.5_ level of less than 30 μg/m^3^. It is likely inappropriate to compare among studies for the exposure threshold because study populations, study regions and methods varied among studies. For example, the US-based Health Professionals Follow-up Study consisted of male healthcare professionals [38] and the Danish Nurse cohort study comprised female nurses [36].

Our study suggested from shapes of exposure-response relation between PM_2.5_ exposure and stroke risk that men were more prone to a higher risk than women (Fig. S VII, (B) and (C)). The slope of positive association was steeper in men than in women for exposing to high PM_2.5_ concentrations. This observation was consistent with previous studies, in which the association between long-term PM_2.5_ exposure and stroke appeared stronger in men than in women [41, 42]. However, our stratified analysis should be considered exploratory, as it was not prior defined, and the interaction effect between sex and PM_2.5_ levels was not statistically significant.

The mechanisms behind the association between long-term exposure to PM_2.5_ and the occurrence of stroke are not fully known. Several proposed pathways might explain our observation of the increased incidence of ischemic stroke associated with elevated PM_2.5_ exposure. Experimental studies have shown that chronic PM exposure may elicit adverse biological responses such as systemic inflammation and oxidative stress, which could result in endothelial dysfunction, development and progression of atherosclerosis, impaired metabolism, and enhanced coagulation/thrombosis [1, 2]. Atherosclerosis is one of the main pathological mechanisms of developing ischemic stroke. Animal studies have shown that chronic exposures to PM_2.5_ enhanced the acceleration of atherosclerosis and plaque vulnerability [43, 44]. Human mechanistic studies also supported the experimental findings. In analyses of Multi-Ethnic Study of Atherosclerosis and Air Pollution, long-term exposure to PM_2.5_ was associated with decreased endothelial function and increased carotid artery intima-medial thickness [45, 46].

In line with the observation of a systematic review [18], our analysis showed a positive association between an increase in annual mean temperature and risk of ischemic stroke (Table 2, Fig. S V). In the time-varying Cox models, the HR of ischemic stroke associated with PM_2.5_ reduced appreciably after adjusting for yearly mean temperature, and the temperature associations also attenuated to borderline significant trends after adjusting for PM_2.5_ (Table S IV). Our data showed that the annual mean PM_2.5_ levels were positively associated with temperature, and the correlation of PM_2.5_ with temperature was stronger than that with other pollutants (Fig. S II). The reduction in HRs associated with PM_2.5_ or temperature was not likely due to multicollinearity. The observed association between time-dependent PM_2.5_ concentrations and stroke could be partly explained by temperature, and vice versa. It is worth noting that the correlation between PM_2.5_ concentrations and temperature varies by several factors such as time scales, geographic regions and components of PM_2.5_. Fu et al. reported the correlation between PM_2.5_ levels and temperature was positive at the daily scale, but was negative at the monthly scale in China [47]. A multiple linear regression model used to analyze 11-year records in the US revealed positive relations between ambient PM_2.5_ concentrations and temperature with varied strengths across regions [48]. Further studies are needed to clarify the role of temperature in the association between PM_2.5_ exposures and incidence of stroke in the long-term settings.

There are limitations to this study. First, the NHI claims data did not provide information on body mass index and lifestyles such as physical activity, alcohol consumption, diet, smoking status. The information on traffic noise levels was also unavailable. We were unable to evaluate whether these factors are associated with air pollution exposures and the risk of developing stroke. Second, misclassification of exposure might occur because data on indoor air pollution and personal daily-activity patterns were unavailable. However, personal daily activity patterns probably have minor impact on the associations in long-term settings. Third, we used PM_2.5_ levels measured at the city/township/district level as a proxy for individuals’ exposure at their residence. In addition, we used the place of registered residence to assign the exposure level for cohort members. This would also result in exposure misclassification in subjects who had jobs or residence not in areas of their registered residence. However, a recent validation study showed that the registered residence was consistent with current residence in 85% of population in Taiwan [35]. Furthermore, both of our sensitivity analyses (Tables S V and S VI) revealed similar and consistent associations to those in the main analysis (Table 2), suggesting that the misclassification of residence probably did not impact substantially our main results. Fourth, study subjects included in our analysis were more likely to reside in areas of high urbanization and income level than were those excluded from our data analysis. Therefore, the study cohort may not be representative to entire population in Taiwan with regards to the social-economic status. Fifth, approximately 98% of the NHI beneficiaries are Taiwanese residents, who are predominately ethnic Chinese. Results of this study may not apply to other ethnic groups.

## Conclusions

Using a population cohort of adults in Taiwan, we found an increased risk of developing ischemic stroke associated with long-term exposure to PM_2.5_. The association with high PM_2.5_ concentrations remained significant after adjustment for other air pollutants and temperature. Results derived from the present study broadens the scope of stroke prevention to include measures aiming to improve air quality applying PM_2.5_ as a key variable. Further studies are needed to clarify if there is a threshold level of long-term PM_2.5_ exposure associated with the risk of developing ischemic stroke.

## Supplementary Information

**Additional file 1.** Online Tables S I –S VI. Online Figure S I –Figure S VII.

## Data Availability

The data that support the findings of this study are available from Health and Welfare Data Science Center, Ministry of Health Welfare, Taiwan, but restrictions apply to the availability of these data, which were used under license for the current study, and so are not publicly available. Data are however available from the authors upon reasonable request and with permission of Health and Welfare Data Science Center, Ministry of Health Welfare, Taiwan.

## Abbreviations

CIs: 95% confidence intervals
CO: Carbon monoxide
HRs: Hazard ratios
ICD-9-CM: International Classification of Diseases, 9th Revision, Clinical Modification IQR Interquartile range
NHI: National Health Insurance Program
NO_2_: Nitrogen dioxide
NO_x_: Nitrogen oxides
O_3_: Ozone
PM_2.5_: Particulate matter with an aerodynamic diameter of 2.5 μm or less
PM_10_: Particulate matter with an aerodynamic diameter of 10 μm or less
SO_2_: Sulfur dioxide
