# Supplementary material for: A cohort study evaluating the risk of stroke associated with long-term exposure to ambient fine particulate matter in Taiwan
Source: Environ Health. 2022 Apr 19;21:43. doi: 10.1186/s12940-022-00854-y (PMC9017007; doi:10.1186/s12940-022-00854-y)
Supplement: Supplementary file 1 — Additional file 1. Online Tables S I –S VI. Online Figure S I –Figure S VII. [file 12940_2022_854_MOESM1_ESM.docx]

**SUPPLEMENTAL MATERIALS**

**A cohort study evaluating the risk of stroke associated with long-term exposure to ambient fine particulate matter in Taiwan**

Pei-Chun Chen, PhD, MS^1*^, Fung-Chang Sung, PhD, MPH^2,3,4*^, Chih-Hsin Mou, MS^3^, Chao W. Chen, PhD^5^, Shan P. Tsai, PhD^6^, Dennis H. P. Hsieh, PhD^7^, Chung Y. Hsu, PhD, MD^8^

^1^Department of Public Health, China Medical University College of Public Health, Taichung, Taiwan

^2^Department of Health Services Administration, China Medical University College of Public Health, Taichung, Taiwan

^3^Management Office for Health Data, China Medical University Hospital, Taichung, Taiwan

^4^Department of Food Nutrition and Health Biotechnology, Asia University, Taichung, Taiwan

^5^University of Maryland Global Campus, Adelphi, MD, USA

^6^School of Public Health, Texas A&M University, College Station, Texas, USA

^7^Department of Environmental Toxicology, University of California at Davis, California USA

^8^Graduate Institute of Biomedical Sciences, China Medical University College of Public Health, Taichung, Taiwan

| **Table S I.** Descriptive characteristics of the population included in and excluded from the study cohort at baseline in 2011 | | | | | |
| --- | --- | --- | --- | --- | --- |
|  | Included in the study cohort  n=1,362,284 | | Excluded from the study cohort  n=1,817,343 | | Standardized mean difference^c^ |
| Variable | n | % | n | % |  |
| Age, year |  |  |  |  |  |
| 20-44 | 735384 | 54.0 | 966440 | 53.2 | 0.016 |
| 45-64 | 495520 | 36.4 | 650472 | 35.8 | 0.012 |
| 65-74 | 82146 | 6.0 | 120653 | 6.6 | 0.025 |
| 75+ | 49234 | 3.6 | 79778 | 4.4 | 0.040 |
| Mean (SD) | 44.0 | (15.2) | 44.4 | (15.6) | 0.026 |
| Men | 659112 | 48.4 | 908023 | 50.0 | 0.032 |
| Urbanization level |  |  |  |  |  |
| 1 (most urbanized) | 359435 | 26.4 | 340162 | 18.7 | 0.184 |
| 2 | 606694 | 44.5 | 376259 | 20.7 | 0.526 |
| 3 | 232680 | 17.1 | 429373 | 23.6 | 0.163 |
| 4-7 | 163475 | 12.0 | 671549 | 37.0 | 0.606 |
| Income^a^, New Taiwan dollars |  |  |  |  |  |
| <=272,470 | 432566 | 31.8 | 887535 | 48.8 | 0.354 |
| 272,471-273,351 | 369701 | 27.1 | 235859 | 13.0 | 0.359 |
| 273,351-311,566 | 338977 | 24.9 | 494185 | 27.2 | 0.053 |
| >311,566 | 221040 | 16.2 | 199764 | 11.0 | 0.153 |
| Comorbidity^b^ |  |  |  |  |  |
| Diabetes | 80181 | 5.9 | 115730 | 6.4 | 0.020 |
| Hypertension | 171447 | 12.6 | 245323 | 13.5 | 0.027 |
| Hyperlipidemia | 113536 | 8.3 | 147867 | 8.1 | 0.007 |
| Coronary artery disease | 42894 | 3.2 | 62238 | 3.4 | 0.010 |
| Chronic obstructive pulmonary disease | 55700 | 4.1 | 77153 | 4.3 | 0.008 |
| Atrial fibrillation | 4054 | 0.3 | 5904 | 0.3 | 0.005 |
| Abbreviations: n, number of study subjects; SD, standard deviation.  ^a^per Capita Disposable Income.  ^b^Patients with at least one inpatient or two outpatient claims with a diagnosis of the comorbid conditions in two years before the index date.  ^c^A standardized mean difference < 0.1 is typically considered as adequate balance in variables between groups. | | | | | |

Table S II. Distributions of annual mean levels of temperature, humidity and air pollutants assigned for each subject by calendar year, 2010-2015, Taiwan

| Year | Number of subjects with missing values (%) | Number of subjects | Mean | Standard deviation | Median | Q1 | Q3 | Minimum | Maximum | IQR |
| --- | --- | --- | --- | --- | --- | --- | --- | --- | --- | --- |
| 2010 |  |  |  |  |  |  |  |  |  |  |
| Temperature, ℃ | 38583 (2.8%) | 1323701 | 23.6 | 1.6 | 23.6 | 23.1 | 24.3 | 16.1 | 25.9 | 1.2 |
| Relative humidity, % | 38583 (2.8%) | 1323701 | 75.0 | 3.9 | 74.9 | 72.4 | 76.5 | 69.4 | 90.3 | 4.1 |
| NO_2_, ppb | 0 (0%) | 1362284 | 20.2 | 6.6 | 21.3 | 16.0 | 25.0 | 2.4 | 33.5 | 9.0 |
| SO_2,_ ppb | 0 (0%) | 1362284 | 4.6 | 2.0 | 4.2 | 3.3 | 5.6 | 1.9 | 11.2 | 2.4 |
| PM_2.5,_ μg/m^3^ | 0 (0%) | 1362284 | 30.4 | 7.2 | 28.2 | 27.0 | 36.6 | 12.6 | 45.0 | 9.6 |
| NO_x_, ppb | 0 (0%) | 1362284 | 30.3 | 16.3 | 28.2 | 20.1 | 34.7 | 4.1 | 85.4 | 14.5 |
| Warm-season O_3_, ppb | 17714 (1.3%) | 1344570 | 46.0 | 5.2 | 46.4 | 42.8 | 49.2 | 31.8 | 58.7 | 6.5 |
| CO, ppm | 0 (0%) | 1362284 | 0.6 | 0.3 | 0.6 | 0.5 | 0.7 | 0.2 | 1.6 | 0.2 |
| 2011 |  |  |  |  |  |  |  |  |  |  |
| Temperature, ℃ | 46225 (3.4%) | 1316059 | 23.2 | 1.4 | 23.2 | 22.8 | 23.8 | 16.6 | 25.1 | 1.0 |
| Relative humidity, % | 46225 (3.4%) | 1316059 | 75.0 | 4.4 | 74.4 | 72.6 | 76.9 | 67.7 | 94.0 | 4.3 |
| NO_2_, ppb | 7855 (0.6%) | 1354429 | 19.0 | 6.4 | 19.1 | 14.9 | 23.0 | 2.1 | 34.6 | 8.1 |
| SO_2,_ ppb | 7855 (0.6%) | 1354429 | 4.1 | 1.7 | 3.7 | 3.1 | 4.7 | 1.7 | 10.1 | 1.6 |
| PM_2.5,_ μg/m^3^ | 7855 (0.6%) | 1354429 | 31.1 | 8.0 | 29.6 | 24.5 | 36.6 | 12.7 | 49.2 | 12.1 |
| NO_x_, ppb | 7855 (0.6%) | 1354429 | 27.2 | 14.6 | 24.7 | 18.4 | 30.6 | 3.9 | 78.5 | 12.2 |
| Warm-season O_3_, ppb | 25455 (1.9%) | 1336829 | 48.0 | 5.7 | 47.8 | 45.3 | 51.8 | 30.4 | 61.3 | 6.5 |
| CO, ppm | 9128 (0.7%) | 1353156 | 0.6 | 0.3 | 0.5 | 0.4 | 0.7 | 0.2 | 1.6 | 0.2 |
| 2012 |  |  |  |  |  |  |  |  |  |  |
| Temperature, ℃ | 74321 (5.5%) | 1287963 | 23.5 | 1.4 | 23.5 | 22.9 | 24.2 | 16.8 | 25.8 | 1.3 |
| Relative humidity, % | 74321 (5.5%) | 1287963 | 75.8 | 4.3 | 75.8 | 73.2 | 77.3 | 68.8 | 94.2 | 4.1 |
| NO_2_, ppb | 36274 (2.7%) | 1326010 | 18.0 | 6.1 | 18.1 | 14.3 | 21.6 | 1.8 | 32.6 | 7.4 |
| SO_2,_ ppb | 36274 (2.7%) | 1326010 | 3.6 | 1.5 | 3.1 | 2.7 | 4.2 | 1.2 | 8.9 | 1.5 |
| PM_2.5,_ μg/m^3^ | 36274 (2.7%) | 1326010 | 27.8 | 7.3 | 25.5 | 23.8 | 32.4 | 11.5 | 45.6 | 8.7 |
| NO_x_, ppb | 36274 (2.7%) | 1326010 | 25.9 | 14.1 | 24.0 | 17.2 | 29.9 | 3.3 | 73.9 | 12.7 |
| Warm-season O_3_, ppb | 53693 (3.9%) | 1308591 | 46.7 | 5.5 | 47.5 | 43.5 | 50.8 | 33.4 | 59.1 | 7.4 |
| CO, ppm | 37606 (2.8%) | 1324678 | 0.6 | 0.3 | 0.5 | 0.4 | 0.7 | 0.2 | 1.6 | 0.2 |
| 2013 |  |  |  |  |  |  |  |  |  |  |
| Temperature, ℃ | 126534 (9.3%) | 1235750 | 23.7 | 1.4 | 23.7 | 23.3 | 24.1 | 17.3 | 26.2 | 0.8 |
| Relative humidity, % | 126534 (9.3%) | 1235750 | 74.7 | 4.3 | 74.0 | 72.2 | 76.1 | 69.9 | 94.1 | 3.9 |
| NO_2_, ppb | 88829 (6.5%) | 1273455 | 18.0 | 6.1 | 18.0 | 14.5 | 21.6 | 1.3 | 32.4 | 7.1 |
| SO_2,_ ppb | 88829 (6.5%) | 1273455 | 3.8 | 1.4 | 3.5 | 2.8 | 4.6 | 1.2 | 8.5 | 1.9 |
| PM_2.5,_ μg/m^3^ | 88829 (6.5%) | 1273455 | 29.4 | 7.1 | 28.5 | 25.8 | 34.1 | 11.8 | 43.7 | 8.3 |
| NO_x_, ppb | 88829 (6.5%) | 1273455 | 25.3 | 13.6 | 22.6 | 17.5 | 29.0 | 2.4 | 73.4 | 11.5 |
| Warm-season O_3_, ppb | 126831 (9.3%) | 1235453 | 45.9 | 5.3 | 46.3 | 44.4 | 49.4 | 31.8 | 55.3 | 5.0 |
| CO, ppm | 90260 (6.6%) | 1272024 | 0.6 | 0.3 | 0.5 | 0.4 | 0.7 | 0.2 | 1.6 | 0.2 |
| 2014 |  |  |  |  |  |  |  |  |  |  |
| Temperature, ℃ | 155647 (11.4%) | 1206637 | 23.8 | 1.4 | 23.9 | 23.2 | 24.5 | 17.5 | 25.9 | 1.2 |
| Relative humidity, % | 155647 (11.4%) | 1206637 | 73.6 | 4.2 | 73.0 | 71.6 | 74.7 | 67.1 | 92.9 | 3.1 |
| NO_2_, ppb | 118142 (8.7%) | 1244142 | 18.1 | 6.2 | 18.3 | 14.8 | 22.1 | 1.6 | 33.8 | 7.3 |
| SO_2,_ ppb | 118142 (8.7%) | 1244142 | 3.7 | 1.2 | 3.5 | 2.9 | 4.2 | 1.3 | 8.3 | 1.4 |
| PM_2.5,_ μg/m^3^ | 118142 (8.7%) | 1244142 | 24.4 | 5.1 | 24.3 | 21.0 | 28.9 | 9.9 | 34.4 | 7.9 |
| NO_x_, ppb | 118142 (8.7%) | 1244142 | 25.4 | 13.7 | 23.0 | 17.7 | 29.1 | 3.0 | 74.7 | 11.4 |
| Warm-season O_3_, ppb | 135102 (9.9%) | 1227182 | 46.3 | 5.0 | 46.9 | 45.0 | 49.4 | 31.5 | 57.6 | 4.5 |
| CO, ppm | 119646 (8.8%) | 1242638 | 0.6 | 0.3 | 0.5 | 0.4 | 0.7 | 0.2 | 1.6 | 0.2 |
| 2015 |  |  |  |  |  |  |  |  |  |  |
| Temperature, ℃ | 162459 (11.9%) | 1199825 | 24.1 | 1.5 | 24.1 | 23.6 | 25.1 | 17.5 | 26.3 | 1.5 |
| Relative humidity, % | 162459 (11.9%) | 1199825 | 74.6 | 4.3 | 73.7 | 72.0 | 76.6 | 69.3 | 93.6 | 4.6 |
| NO_2,_ ppb | 125327 (9.2%) | 1236957 | 17.0 | 5.8 | 17.3 | 14.0 | 20.5 | 2.0 | 30.9 | 6.5 |
| SO_2,_ ppb | 125327 (9.2%) | 1236957 | 3.4 | 1.0 | 3.4 | 2.8 | 3.8 | 1.3 | 7.3 | 1.0 |
| PM_2.5,_ μg/m^3^ | 125327 (9.2%) | 1236957 | 21.1 | 4.5 | 20.7 | 18.3 | 24.4 | 9.5 | 32.1 | 6.1 |
| NO_x_, ppb | 125327 (9.2%) | 1236957 | 23.5 | 12.3 | 21.4 | 16.7 | 27.9 | 3.6 | 68.5 | 11.2 |
| Warm-season O_3_, ppb | 177803 (13.1%) | 1184481 | 43.9 | 5.1 | 45.7 | 41.1 | 47.2 | 29.2 | 54.4 | 6.0 |
| CO, ppm | 126843 (9.3%) | 1235441 | 0.6 | 0.3 | 0.5 | 0.4 | 0.7 | 0.2 | 1.6 | 0.2 |

Abbreviations: CO, carbon monoxide; NO_2_, nitrogen dioxide; PM_2.5_, particulate matter of ≤2.5 µm in diameter; Q1, the first quartile; Q3, the third quartile; SO_2_, Sulfur dioxide.

| **Table S III.** Descriptive characteristics of study subjects in the study cohort included in the main analysis of PM_2.5_ exposure and those with temperature data | | | | |
| --- | --- | --- | --- | --- |
|  | Subjects for main analysis (PM_2.5_ exposure), n=1,362,284 | | Subjects with temperature data, n=1,323,701 | |
| Characteristics at baseline | n | % | n | % |
| Age, years |  |  |  |  |
| 20-44 | 735384 | 54.0 | 715519 | 54.1 |
| 45-64 | 495520 | 36.4 | 480917 | 36.3 |
| 65-74 | 82146 | 6.0 | 79558 | 6.0 |
| 75+ | 49234 | 3.6 | 47707 | 3.6 |
| Mean (SD) | 44.0 | (15.2) | 43.9 | (15.2) |
| Men | 659112 | 48.4 | 640696 | 48.4 |
| Urbanization level |  |  |  |  |
| 1 (most urbanized) | 359435 | 26.4 | 341721 | 25.8 |
| 2 | 606694 | 44.5 | 585825 | 44.3 |
| 3 | 232680 | 17.1 | 232680 | 17.6 |
| 4-7 | 163475 | 12.0 | 163475 | 12.4 |
| Income^a^, New Taiwan dollars |  |  |  |  |
| <=272470 | 432566 | 31.8 | 432566 | 32.7 |
| 272471-273351 | 369701 | 27.1 | 348832 | 26.4 |
| 273351-311566 | 338977 | 24.9 | 338977 | 25.6 |
| >311566 | 221040 | 16.2 | 203326 | 15.4 |
| Comorbidity^b^ |  |  |  |  |
| Diabetes | 80181 | 5.9 | 77774 | 5.9 |
| Hypertension | 171447 | 12.6 | 166564 | 12.6 |
| Hyperlipidemia | 113536 | 8.3 | 109821 | 8.3 |
| Coronary artery disease | 42894 | 3.2 | 41591 | 3.1 |
| Chronic obstructive pulmonary disease | 55700 | 4.1 | 54072 | 4.1 |
| Atrial fibrillation | 4054 | 0.3 | 3944 | 0.3 |
| Abbreviations: n, number of study subjects; PM_2.5_, particulate matter of ≤2.5µm in diameter; SD, standard deviation.  ^a^per Capita Disposable Income.  ^b^Patients with at least one inpatient or two outpatient claims with a diagnosis of the comorbid conditions within two years before the index date. | | | | |

**Table S IV.** Hazard ratios (95% confidence intervals) of stroke in association with air pollutants as time-dependent covariates (every IQR increase) in the two-exposure models

|  | Single-exposure model^a^ |  | Two-exposure models^a^, additionally adjusted for | | | | | | |
| --- | --- | --- | --- | --- | --- | --- | --- | --- | --- |
|  |  |  | PM_2.5,_ μg/m^3^ | SO_2,_ ppb | NO_2_, ppb | Warm-season O_3_, ppb | CO, ppm | Temperature℃ |  |
| Total Stroke |  |  |  |  |  |  |  |  |  |
| PM_2.5,_ μg/m^3^ | 1.03 (1.01-1.05) |  | -- | 1.00 (0.97-1.03) | 1.00 (0.97-1.03) | 1.04 (1.02-1.07) | 1.02 (1.00-1.04)^b^ | 0.99 (0.96-1.02) |  |
| SO_2,_ ppb | 1.03 (1.02-1.05) |  | 1.03 (1.01-1.06) | -- | 1.02 (0.99-1.05) | 1.03 (1.01-1.05) | 1.02 (1.01-1.04) | 1.02 (1.00-1.05)^b^ |  |
| NO_2_, ppb | 1.04 (1.02-1.07) |  | 1.05 (1.01-1.08) | 1.03 (1.00-1.07)^b^ | -- | 1.03 (1.01-1.06) | 1.01 (0.98-1.05) | 1.03 (1.00-1.07)^b^ |  |
| Warm-season O_3_, ppb | 0.97 (0.96-0.99) |  | 0.96 (0.95-0.98) | 0.98 (0.96-0.99) | 0.98 (0.96-0.99) | -- | 0.98 (0.96-1.00) | 0.97 (0.96-0.99) |  |
| CO, ppm | 1.03 (1.01-1.04) |  | 1.02 (1.01-1.04) | 1.02 (1.01-1.04) | 1.02 (1.00-1.04)^b^ | 1.02 (1.01-1.04) | -- | 1.02 (1.01-1.04) |  |
| Temperature, ℃ | 1.02 (1.01-1.03) |  | 1.02 (1.00-1.04) | 1.01 (1.00-1.03)^b^ | 1.01 (1.00-1.03)^b^ | 1.02 (1.01-10.3) | 1.02 (1.01-1.03) | -- |  |
| Ischemic stroke |  |  |  |  |  |  |  |  |  |
| PM_2.5,_ μg/m^3^ | 1.05 (1.03-1.08) |  | -- | 1.02 (0.99-1.06) | 1.03 (1.00-1.06)^b^ | 1.07 (1.04-1.09) | 1.05 (1.02-1.07) | 1.01 (0.98-1.05) |  |
| SO_2,_ ppb | 1.05 (1.03-1.07) |  | 1.04 (1.01-1.07) | -- | 1.04 (1.01-1.07) | 1.05 (1.03-1.07) | 1.04 (1.02-1.06) | 1.04 (1.01-1.07) |  |
| NO_2_, ppb | 1.05 (1.03-1.08) |  | 1.05 (1.01-1.09) | 1.03 (0.99-1.07) | -- | 1.05 (1.02-1.08) | 1.04 (1.00-1.09)^b^ | 1.03 (1.00-1.07)^b^ |  |
| Warm-season O_3_, ppb | 0.99 (0.97-1.01) |  | 0.97 (0.95-0.99) | 1.00 (0.98-1.01) | 1.00 (0.98-1.01) | -- | 1.00 (0.98-1.02) | 0.99 (0.97-1.01) |  |
| CO, ppm | 1.03 (1.01-1.04) |  | 1.02 (1.01-1.04) | 1.02 (1.00-1.03) | 1.01 (0.99-1.03) | 1.02 (1.00-1.04) | -- | 1.02 (1.00-1.03)^b^ |  |
| Temperature, ℃ | 1.03 (1.02-1.04) |  | 1.02 (1.00-1.05) | 1.02 (1.00-1.04) | 1.02 (1.01-1.04) | 1.03 (0.97-1.01) | 1.03 (1.01-1.04) | -- |  |
| Hemorrhagic stroke |  |  |  |  |  |  |  |  |  |
| PM_2.5,_ μg/m^3^ | 0.94 (0.91-0.98) |  | -- | 0.93 (0.88-0.99) | 0.91 (0.86-0.97) | 0.97 (0.93-1.01) | 0.93 (0.89-0.97) | 0.92 (0.86-0.98) |  |
| SO_2,_ ppb | 0.97 (0.94-1.01) |  | 1.00 (0.95-1.05) | -- | 0.95 (0.89-1.00) | 0.96 (0.92-0.99) | 0.96 (0.92-0.99) | 0.97 (0.93-1.03) |  |
| NO_2_, ppb | 1.01 (0.97-1.06) |  | 1.06 (0.99-1.13) | 1.05 (0.98-1.13) | -- | 0.99 (0.95-1.04) | 0.94 (0.97-1.01) | 1.04 (0.97-1.11) |  |
| Warm-season O_3_, ppb | 0.93 (0.90-0.96) |  | 0.93 (0.90-0.97) | 0.92 (0.89-0.95) | 0.93 (0.90-0.96) | -- | 0.93 (0.90-0.96) | 0.93 (0.90-0.96) |  |
| CO, ppm | 1.03 (1.00-1.05) |  | 1.03 (1.01-1.06) | 1.04 (1.01-1.06) | 1.06 (1.01-1.10) | 1.01 (0.98-1.05) | -- | 1.05 (1.01-1.08) |  |
| Temperature, ℃ | 0.99 (0.96-1.01) |  | 1.02 (0.98-1.06) | 0.99 (0.96-1.02) | 0.98 (0.95-1.01) | 0.99 (0.96-1.01) | 0.98 (0.96-1.01) | -- |  |

Abbreviations: CO, carbon monoxide; IQR, interquartile range; NO_2_, nitrogen dioxide; NO_x_, nitrogen oxides;

PM_2.5_, particulate matter of ≤2.5µm in diameter; SO_2_, sulfur dioxide.

^a^The model was adjusted for age, sex, income, and urbanization level.

^b^Borderline significance (*P*>0.05)

| **Table S V.** Sensitivity analysis: hazard ratios (95% confidence intervals) for the association between every interquartile-range increase in air pollutant levels and incident stroke in which the exposure level was assigned to based on estimated current residence | | | | | |
| --- | --- | --- | --- | --- | --- |
|  | Number of study subjects included in the analysis | 1-year exposure at baseline | | Air pollution exposure as a time-varying covariate | |
|  |  | Model 1^a^ | Model 2 (Main model)^b^ | Model 1^a^ | Model 2 (Main model)^b^ |
| Total Stroke |  |  |  |  |  |
| PM_2.5,_ μg/m^3^ | 617783 | 1.09 (1.06-1.13) | 1.06 (1.01-1.11) | 1.10 (1.07-1.13) | 1.05 (1.02-1.08) |
| SO_2_, ppb | 617783 | 1.04 (1.01-1.08) | 1.01 (0.97-1.05) | 1.05 (1.02-1.07) | 1.02 (1.00-1.05) |
| NO_x_, ppb | 617783 | 0.97 (0.95-0.99) | 1.04 (1.01-1.08) | 0.97 (0.96-0.99) | 1.04 (1.02-1.07) |
| NO_2_, ppb | 617783 | 0.94 (0.91-0.98) | 1.04 (0.99-1.09) | 0.95 (0.93-0.98) | 1.05 (1.01-1.08) |
| Warm-season O_3_^c^, ppb | 610441 | 1.05 (1.02-1.09) | 0.99 (0.95-1.02) | 1.04 (1.02-1.07) | 0.98 (0.96-1.00) |
| CO, ppm | 617783 | 0.97 (0.95-0.99) | 1.04 (1.01-1.07) | 0.98 (0.96-0.99) | 1.03 (1.01-1.05) |
| Temperature, ℃ | 602224 | 1.05 (1.03-1.08) | 1.03 (1.00-1.06) | 1.05 (1.03-1.07) | 1.03 (1.01-1.05) |
| Relative humidity, % | 602224 | 1.02 (0.99-1.05) | 1.00 (0.96-1.03) | 1.01 (0.99-1.03) | 0.99 (0.97-1.02) |
| Ischemic stroke |  |  |  |  |  |
| PM_2.5,_ μg/m^3^ | 617783 | 1.13 (1.09-1.18) | 1.10 (1.04-1.16) | 1.14 (1.10-1.17) | 1.09 (1.05-1.13) |
| SO_2_, ppb | 617783 | 1.06 (1.03-1.10) | 1.03 (0.99-1.07) | 1.07 (1.04-1.10) | 1.05 (1.02-1.08) |
| NO_x_, ppb | 617783 | 0.97 (0.94-0.99) | 1.04 (1.00-1.08) | 0.97 (0.95-0.99) | 1.05 (1.02-1.07) |
| NO_2_, ppb | 617783 | 0.95 (0.91-0.99) | 1.05 (1.00-1.12) | 0.96 (0.94-0.99) | 1.07 (1.03-1.11) |
| Warm-season O_3_^c^, ppb | 610441 | 1.08 (1.04-1.13) | 1.01 (0.97-1.05) | 1.06 (1.04-1.09) | 1.00 (0.97-1.02) |
| CO, ppm | 617783 | 0.96 (0.94-0.99) | 1.03 (1.00-1.07) | 0.97 (0.95-0.99) | 1.03 (1.01-1.05) |
| Temperature, ℃ | 602224 | 1.08 (1.05-1.11) | 1.05 (1.02-1.09) | 1.08 (1.05-1.10) | 1.05 (1.03-1.07) |
| Relative humidity, % | 602224 | 1.01 (0.97-1.04) | 0.98 (0.94-1.02) | 0.99 (0.97-1.02) | 0.98 (0.95-1.01) |
| Hemorrhagic stroke |  |  |  |  |  |
| PM_2.5,_ μg/m^3^ | 617783 | 0.96 (0.89-1.04) | 0.91 (0.82-1.01) | 0.95 (0.90-1.01) | 0.92 (0.86-0.98) |
| SO_2_, ppb | 617783 | 0.96 (0.90-1.04) | 0.93 (0.86-1.01) | 0.96 (0.91-1.01) | 0.93 (0.88-0.99) |
| NO_x_, ppb | 617783 | 0.99 (0.94-1.04) | 1.05 (0.98-1.12) | 0.98 (0.94-1.02) | 1.04 (0.99-1.09) |
| NO_2_, ppb | 617783 | 0.93 (0.86-1.00) | 0.99 (0.89-1.10) | 0.92 (0.88-0.98) | 0.98 (0.91-1.05) |
| Warm-season O_3_^c^, ppb | 610441 | 0.95 (0.88-1.02) | 0.90 (0.83-0.97) | 0.96 (0.91-1.01) | 0.92 (0.87-0.97) |
| CO, ppm | 617783 | 1.00 (0.95-1.05) | 1.07 (1.00-1.14) | 0.99 (0.96-1.03) | 1.04 (1.00-1.09) |
| Temperature, ℃ | 602224 | 0.97 (0.93-1.02) | 0.95 (0.90-1.00) | 0.97 (0.94-1.01) | 0.95 (0.92-0.99) |
| Relative humidity, % | 602224 | 1.06 (1.00-1.13) | 1.05 (0.98-1.13) | 1.04 (1.00-1.09) | 1.04 (0.99-1.09) |

Abbreviations: CO, carbon monoxide; NO_2_, nitrogen dioxide; NO_x_, nitrogen oxides; O_3_, ozone; PM_2.5_, particulate matter with diameter ≤2.5 μm; SO_2_, sulfur dioxide.

^a^Model 1 was adjusted for age and sex.

^b^Model 2 was adjusted for age, sex, income, and urbanization level.

^c^April-October daily maximum 8-hour ozone concentrations.

| **Table S VI.** Sensitivity analysis: hazard ratios (95% confidence intervals) for the association between every interquartile-range increase in air pollutant levels and incident stroke excluding people whose location of registered residence was inconsistent with that of their NHI registration | | | | | |
| --- | --- | --- | --- | --- | --- |
|  | Number of study subjects included in the analysis | 1-year exposure at baseline | | Air pollution exposure as a time-varying covariate | |
|  |  | Model 1^a^ | Model 2 (Main model)^b^ | Model 1^a^ | Model 2 (Main model)^b^ |
| Total Stroke |  |  |  |  |  |
| PM_2.5,_ μg/m^3^ | 568633 | 1.06 (1.03-1.09) | 1.03 (0.99-1.07) | 1.07 (1.04-1.09) | 1.02 (1.00-1.05) |
| SO_2_, ppb | 568633 | 1.05 (1.03-1.08) | 1.04 (1.00-1.07) | 1.05 (1.03-1.07) | 1.04 (1.02-1.06) |
| NO_x_, ppb | 568633 | 0.96 (0.94-0.98) | 1.04 (1.01-1.07) | 0.97 (0.95-0.98) | 1.04 (1.02-1.06) |
| NO_2_, ppb | 568633 | 0.94 (0.91-0.96) | 1.03 (0.99-1.08) | 0.95 (0.93-0.97) | 1.05 (1.02-1.08) |
| Warm-season O_3_^c^, ppb | 562290 | 1.04 (1.01-1.07) | 0.98 (0.95-1.01) | 1.03 (1.01-1.05) | 0.97 (0.95-0.99) |
| CO, ppm | 568633 | 0.96 (0.94-0.98) | 1.04 (1.01-1.07) | 0.97 (0.96-0.98) | 1.03 (1.01-1.05) |
| Temperature,℃ | 553952 | 1.04 (1.02-1.06) | 1.01 (0.99-1.04) | 1.04 (1.03-1.06) | 1.01 (0.99-1.03) |
| Relative humidity, % | 553952 | 1.02 (0.99-1.05) | 1.00 (0.97-1.03) | 1.01 (0.99-1.03) | 1.00 (0.98-1.02) |
| Ischemic stroke |  |  |  |  |  |
| PM_2.5,_ μg/m^3^ | 568633 | 1.11 (1.07-1.15) | 1.06 (1.01-1.10) | 1.11 (1.09-1.14) | 1.05 (1.02-1.08) |
| SO_2_, ppb | 568633 | 1.09 (1.06-1.12) | 1.06 (1.02-1.10) | 1.09 (1.06-1.11) | 1.06 (1.04-1.09) |
| NO_x_, ppb | 568633 | 0.95 (0.93-0.98) | 1.04 (1.00-1.07) | 0.96 (0.94-0.98) | 1.04 (10.1-1.06) |
| NO_2_, ppb | 568633 | 0.93 (0.90-0.97) | 1.04 (0.99-1.09) | 0.95 (0.92-0.97) | 1.05 (1.02-1.09) |
| Warm-season O_3_^c^, ppb | 562290 | 1.07 (1.04-1.10) | 1.00 (0.97-1.03) | 1.05 (10.3-1.08) | 0.99 (0.97-1.01) |
| CO, ppm | 568633 | 0.95 (0.93-0.98) | 1.03 (1.00-1.07) | 0.96 (0.94-0.98) | 1.02 (1.00-1.04) |
| Temperature,℃ | 553952 | 1.06 (1.04-1.09) | 1.03 (1.00-1.05) | 1.06 (1.04-1.08) | 1.02 (1.00-1.04) |
| Relative humidity, % | 553952 | 1.02 (0.99-1.04) | 1.00 (0.96-1.03) | 1.01 (0.99-1.03) | 1.00 (0.97-1.02) |
| Hemorrhagic stroke |  |  |  |  |  |
| PM_2.5,_ μg/m^3^ | 568633 | 0.92 (0.86-0.98) | 0.95 (0.88-1.03) | 0.92 (0.88-0.96) | 0.93 (0.88-0.98) |
| SO_2_, ppb | 568633 | 0.94 (0.88-1.00) | 0.96 (0.90-1.03) | 0.94 (0.90-0.98) | 0.96 (0.91-1.01) |
| NO_x_, ppb | 568633 | 0.99 (0.95-1.04) | 1.05 (0.99-1.12) | 1.00 (0.97-1.04) | 1.06 (1.02-1.11) |
| NO_2_, ppb | 568633 | 0.94 (0.88-1.00) | 1.02 (0.93-1.12) | 0.96 (0.91-1.00) | 1.04 (0.97-1.10) |
| Warm-season O_3_^c^, ppb | 562290 | 0.95 (0.90-1.01) | 0.92 (0.86-0.98) | 0.93 (0.90-0.97) | 0.91 (0.87-0.94) |
| CO, ppm | 568633 | 0.99 (0.95-1.04) | 1.05 (0.99-1.11) | 1.00 (0.97-1.03) | 1.04 (1.01-1.09) |
| Temperature,℃ | 553952 | 0.98 (0.94-1.01) | 0.98 (0.93-1.03) | 0.98 (0.96-1.01) | 0.98 (0.95-1.01) |
| Relative humidity, % | 553952 | 1.04 (0.98-1.09) | 1.01 (0.95-1.08) | 1.02 (0.98-1.06) | 1.01 (0.97-1.05) |

Abbreviations: CO, carbon monoxide; NHI, National Health Insurance; NO_2_, nitrogen dioxide; NO_x_, nitrogen oxides; O_3_, ozone; PM_2.5_, particulate matter of ≤2.5µm in diameter; SO_2_, sulfur dioxide.

^a^Model 1 was adjusted for age and sex.

^b^Model 2 was adjusted for age, sex, income, and urbanization level.

^c^April-October daily maximum 8-hour ozone concentrations.

**Figure S I.** Map of study areas, populations and air quality monitoring stations, Taiwan. Areas were administrative subdivisions in terms of city, township, and district defined by local government. The areas excluded from our data analysis are in solid white color. The red dots are sites equipped with air quality monitoring stations in which the measurements of air pollution, temperature and relative humidity were recorded.

**
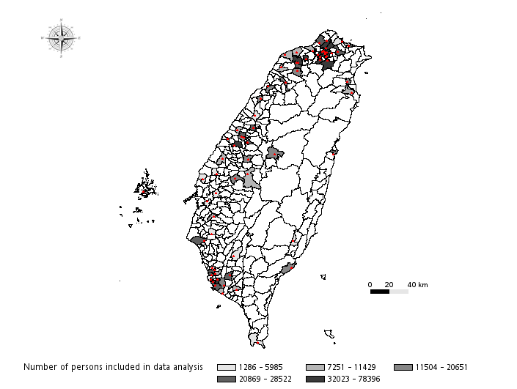
**

**Figure S II.** Spearman correlation coefficients between annul mean levels of environmental exposures from 2010 to 2015. Abbreviations: CO, carbon monoxide; NO_2_, nitrogen dioxide; O_3_, ozone; PM_2.5_, particulate matter of ≤2.5 µm in diameter; SO_2_, Sulfur dioxide; Temp, temperature. **
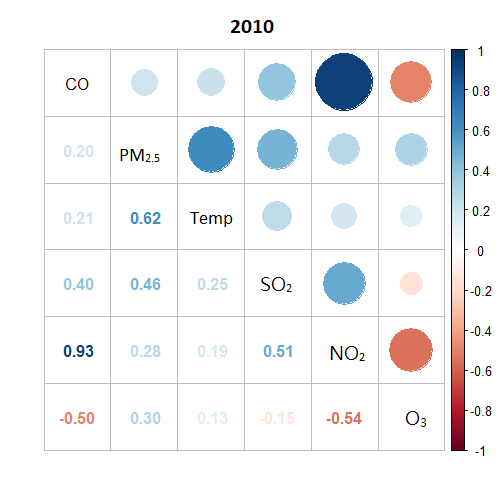

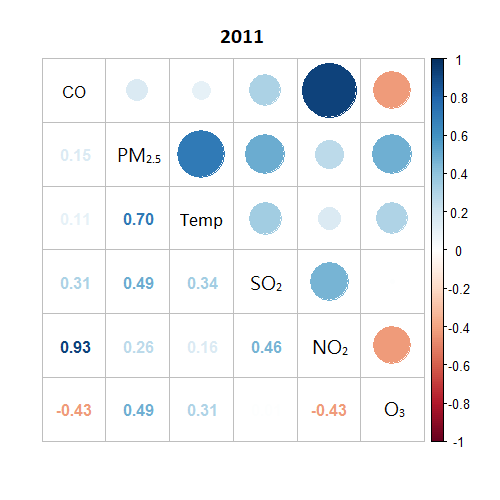

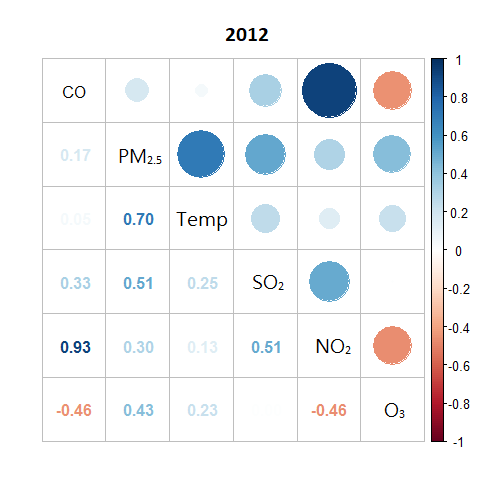

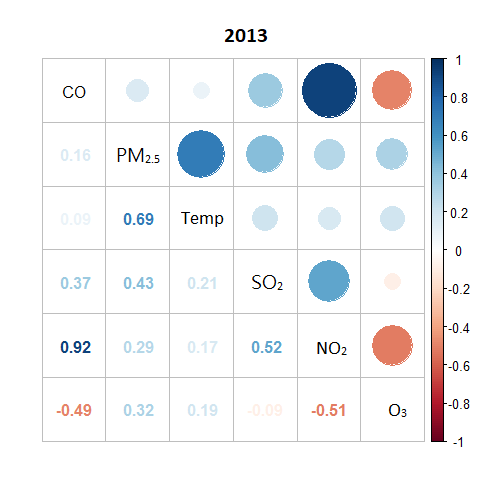

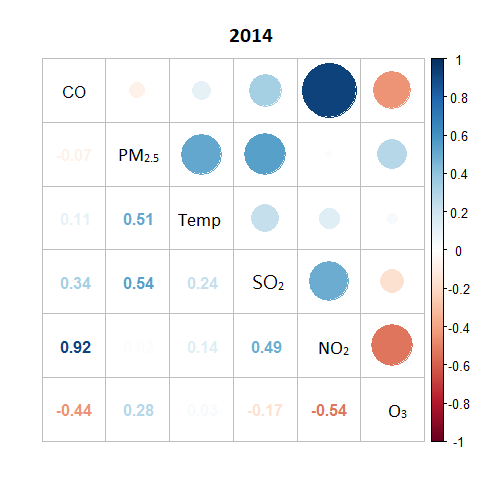

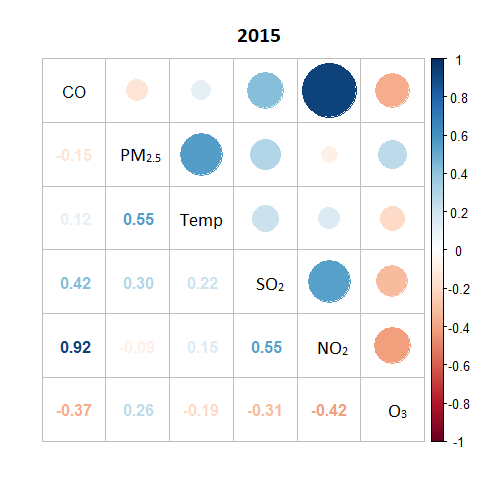
**

**Figure S III.** Dose-response curves estimated with natural cubic spline models for the association between 1-year exposure of PM_2.5_ at baseline and risk of all stroke, ischemic stroke and hemorrhagic stroke. in the main model (A) and the main model with additional adjustment for temperature (B), SO_2_ (C), NO_2_ (D), Warm-season O_3_ (E), and CO (F).

(A) the main model adjusted for age, sex, income, and urbanization level. (B) to (F), the main model with additional adjustment for temperature (B), SO_2_ (C), NO_2_ (D), Warm-season O_3_ (E), and CO (F). The Solid black line indicates adjusted HRs based on restricted cubic spline model with three knots located at the 10th, 50th, and 90th percentiles of the distribution of PM_2.5_ levels. The dashed lines indicates 95% confidence interval. The reference values were set at the median of the distribution of PM_2.5_ concentrations. Abbreviations: CO, carbon monoxide; NO_2_, nitrogen dioxide; PM_2.5_, particulate matter of ≤2.5 µm in diameter.

1. Main model





1. Main model + adjustment for temperature





1. Main model + adjustment for SO_2_





1. Main model + adjustment for NO_2_





1. Main model + adjustment for warm-season O_3_





1. Main model + adjustment for CO





**Figure S IV.** Hazard ratios (HRs) of all stroke, ischemic stroke and hemorrhagic stroke by long-term exposure to PM_2.5_ levels estimated using time-dependent Cox model without (A) and with additional adjustment for temperature (B), SO_2_ (C), NO_2_ (D), Warm-season O_3_ (E), and CO (F).

The main model was adjusted for age, sex, income, and urbanization level. The Solid black line indicates adjusted HRs based on restricted cubic spline model with three knots located at the 10th, 50th, and 90th percentiles of the distribution of PM_2.5_ levels. The dashed lines indicate 95% confidence interval. The median of PM_2.5_ concentration was used as the reference value. Abbreviations: CO, carbon monoxide; NO_2_, nitrogen dioxide; O_3_, ozone; PM_2.5_, particulate matter that is ≤2.5 µm in diameter; SO_2_, Sulfur dioxide.

1. Main model





1. Main model + adjustment for temperature





1. Main model + adjustment for SO_2_





1. Main model + adjustment for NO_2_





1. Model 1 + adjustment for Warm-season O_3_





1. Main model + adjustment for CO





**Figure S V.** Hazard ratios (HRs) of all stroke, ischemic stroke and hemorrhagic stroke by temperature. The Solid black line indicates adjusted HRs based on restricted cubic spline model with three knots located at the 10th, 50th, and 90th percentiles of the distribution of temperaure levels. The dashed lines indicate 95% confidence interval. The median temperature level was used as the reference value. All models were adjsuted for age, sex, income level and urbanization level.

1. Models with 1-year mean temperature at baseline





1. Models with yearly mean temperature as a time-dependent covariate





**Figure S VI.** Hazard ratios (95% confidence intervals) of ischemic stroke (A) and hemorrhagic stroke (B) associated with PM_2.5_ levels by comorbidity in Cox proportional hazards models. All models were adjusted for age, sex, income level and urbanization level. Abbreviations: IQR, interquartile range; PM_2.5_, particulate matter ≤2.5 µm in diameter

**

**

Ischemic stroke

**

**

Hemorrhagic stroke

**Figure S VII.** Hazard ratios (HRs) of all stroke (A), ischemic stroke (B) and hemorrhagic stroke (C) by long-term exposure to PM_2.5_ levels estimated using time-dependent Cox model after controlling for age, sex, income and urbanization by sex.

The Solid black line indicates adjusted HRs based on restricted cubic spline model with three knots located at the 10th, 50th, and 90th percentiles of PM_2.5_ distribution. The median of PM_2.5_ concentration was used as the reference value. P values for interaction between PM_2.5_ and sex: 0.88 for all stroke, 0.43 for ischemic stroke, and 0.24 for hemorragic stroke. Abbreviations: PM_2.5_, particulate matter that is ≤2.5 µm in diameter.

1. All stroke





1. Ischemic stroke





1. Hemorrhagic stroke
